# First-line nanoparticle polymeric micellar paclitaxel with gemcitabine in metastatic pancreatic cancer: a single-arm, prospective, and exploratory study

**DOI:** 10.1093/gastro/goag034

**Published:** 2026-05-01

**Authors:** Nan Lyu, Qianqian Wang, Kuirong Jiang, Dong Xu, Yang Wu, Kai Zhang, Jishu Wei, Jianmin Chen, Feng Guo, Zipeng Lu, Bin Xiao, Guosheng Chen, Junli Wu, Wentao Gao, Yuqi Wang, Fufeng Wang, Min Tu

**Affiliations:** Pancreas Center, The First Affiliated Hospital with Nanjing Medical University, 300 Guangzhou Road, Nanjing, Jiangsu, 210029, China; Pancreas Center, The First Affiliated Hospital with Nanjing Medical University, 300 Guangzhou Road, Nanjing, Jiangsu, 210029, China; Pancreas Center, The First Affiliated Hospital with Nanjing Medical University, 300 Guangzhou Road, Nanjing, Jiangsu, 210029, China; Pancreas Center, The First Affiliated Hospital with Nanjing Medical University, 300 Guangzhou Road, Nanjing, Jiangsu, 210029, China; Pancreas Center, The First Affiliated Hospital with Nanjing Medical University, 300 Guangzhou Road, Nanjing, Jiangsu, 210029, China; Pancreas Center, The First Affiliated Hospital with Nanjing Medical University, 300 Guangzhou Road, Nanjing, Jiangsu, 210029, China; Pancreas Center, The First Affiliated Hospital with Nanjing Medical University, 300 Guangzhou Road, Nanjing, Jiangsu, 210029, China; Pancreas Center, The First Affiliated Hospital with Nanjing Medical University, 300 Guangzhou Road, Nanjing, Jiangsu, 210029, China; Pancreas Center, The First Affiliated Hospital with Nanjing Medical University, 300 Guangzhou Road, Nanjing, Jiangsu, 210029, China; Pancreas Center, The First Affiliated Hospital with Nanjing Medical University, 300 Guangzhou Road, Nanjing, Jiangsu, 210029, China; Pancreas Center, The First Affiliated Hospital with Nanjing Medical University, 300 Guangzhou Road, Nanjing, Jiangsu, 210029, China; Pancreas Center, The First Affiliated Hospital with Nanjing Medical University, 300 Guangzhou Road, Nanjing, Jiangsu, 210029, China; Pancreas Center, The First Affiliated Hospital with Nanjing Medical University, 300 Guangzhou Road, Nanjing, Jiangsu, 210029, China; Pancreas Center, The First Affiliated Hospital with Nanjing Medical University, 300 Guangzhou Road, Nanjing, Jiangsu, 210029, China; Geneseeq Research Institute, Nanjing Geneseeq Technology Inc., Nanjing, Jiangsu, 211800, P. R. China; Geneseeq Research Institute, Nanjing Geneseeq Technology Inc., Nanjing, Jiangsu, 211800, P. R. China; Pancreas Center, The First Affiliated Hospital with Nanjing Medical University, 300 Guangzhou Road, Nanjing, Jiangsu, 210029, China; Pancreas Center, Sir Run Run Hospital, Nanjing Medical University, Nanjing, Jiangsu, 211100, P. R. China

**Keywords:** metastatic pancreatic cancer, chemotherapy, nanoparticle polymeric micellar paclitaxel, transcriptional profiling

## Abstract

**Background:**

Pancreatic cancer is one of the most lethal malignancies, with limited therapeutic options. In this exploratory trial, we aimed to evaluate the efficacy and safety of nanoparticle polymeric micellar paclitaxel (pm-Pac) combined with gemcitabine as first-line treatment for metastatic pancreatic cancer (mPC).

**Methods:**

Twenty-one patients with histologically or cytologically confirmed mPC were enrolled in this study. The primary endpoint was progression-free survival (PFS). Meanwhile, the secondary endpoints included the objective response rate (ORR), overall survival, disease control rate (DCR), duration of response (DOR), and safety of combination therapy.

**Results:**

The median PFS was 7.4 months (95% confidence interval [CI]: 5.4–9.4 months). The ORR and DCR were 52.4% (95% CI: 29.1%–75.7%) and 95.2% (95% CI: 85.3%–100%), respectively. Amongst patients who achieved partial response, the median DOR was 4.8 months (95% CI: 1.5–8.1 months). No treatment-related deaths were reported. Grade 3–4 adverse events (AEs) occurred in 81.0% of patients, with increased γ-glutamyltransferase levels (38.1%), neutropenia (33.3%), and leukocytopenia (28.6%) being the most frequent AEs. Low SERPINB3 and SERPINB4 expression was correlated with prolonged PFS, accompanied by the significant downregulation of gene sets involved in DNA replication, nonsense-mediated mRNA decay, and protein translation in long-PFS tumours. Tumour immune microenvironment analysis revealed that patients with short PFS had increased levels of common lymphoid progenitors and decreased populations of mature B and T lymphocytes.

**Conclusions:**

The combination of pm-Pac and gemcitabine as first-line therapy for mPC exhibited favourable tolerability and clinical efficacy. However, larger randomized–controlled trials are needed to validate these preliminary findings.

**Trial Registration:**

www.chictr.org.cn, ChiCTR2300078861

## Introduction

In recent years, the incidence and mortality rates of pancreatic cancer (one of the most aggressive and lethal forms of cancer) have steadily increased [1–3]. Despite the approval of new drugs, the median survival for pancreatic cancer remains <1 year [4]. In 2025, cancer statistics showed that the 5-year survival rate for pancreatic cancer remained at 13% [1]. Pancreatic cancer is the third-leading cause of cancer-related deaths and it is projected to become the second-most common malignancy by 2030 [5, 6]. Most patients with pancreatic cancer are diagnosed when surgical resection is no longer an option, with 50%–60% presenting metastases at diagnosis [6, 7]. At present, systemic chemotherapy remains the primary treatment for advanced metastatic pancreatic cancer (mPC) [8]. Current domestic and international guidelines recommend NALIRIFOX, FOLFIRINOX, and AG regimens (gemcitabine combined with albumin-bound paclitaxel) as first-line combination chemotherapy options [7, 9–12]. According to clinical trials, FOLFIRINOX and NALIRIFOX regimens exhibit similar and relatively favourable survival outcomes in select patients with mPC (median progression-free survival [mPFS], 6.4–7.4 months; median overall survival [mOS], 11.1 months) [10, 11]. In comparison, the AG regimen shows shorter survival (mPFS, 5.5–5.6 months; mOS, 8.5–9.2 months) [10, 12]. Usually, NALIRIFOX and FOLFIRINOX regimens are recommended for patients with good performance status [8, 10, 11]. However, most patients with advanced pancreatic cancer have poor performance status, leading to the use of the AG regimen. Considering the poor prognosis in most patients and the limited number of available therapeutic regimens, there is a need to explore efficacious and tolerable new treatment approaches and maximize the therapeutic benefits of cytotoxic chemotherapy regimens.

Nanoparticle polymeric micellar paclitaxel (pm-Pac; Shanghai Yizhong Pharmaceutical Co., Ltd) is a novel paclitaxel formulation that is distinct from solvent-based, liposomal, and albumin-bound paclitaxel. pm-Pac comprises paclitaxel encapsulated in monomethoxy polyethylene glycol 2000-polylactic acid (mPEG-PDLLA) nanoparticles by using nanotechnology. The tumour-targeting mechanism of pm-Pac relies on the enhanced permeability and retention effect, in which larger-capillary intercellular spaces in tumours allow nanoparticles to extravasate and accumulate preferentially. The mPEG-PDLLA copolymer forms micelles with a hydrophobic core (solubilizing paclitaxel) and hydrophilic shell, ensuring stability. These micelles are biodegradable and small (∼20 nm), which allows them to evade reticuloendothelial system clearance, thereby enhancing tumour delivery [13, 14]. The pharmacological mechanism of pm-Pac is well established. According to phase I clinical studies in patients with advanced solid tumours, pm-Pac exhibits good safety and tolerability [15]. Moreover, results from phase III clinical studies in non-small cell lung cancer (NSCLC) showed that pm-Pac combined with cisplatin significantly improved the objective response rate (ORR) and prolonged the progression-free survival (PFS) of patients with advanced first-line NSCLC compared with conventional paclitaxel injection combined with cisplatin [16].

Currently, no studies have used pm-Pac combined with gemcitabine to treat advanced mPC. Therefore, in the present study, we aimed to evaluate the clinical efficacy of pm-Pac combined with gemcitabine as first-line treatment for mPC.

## Patients and methods

### Study design

This single-arm, prospective, and exploratory study was designed to evaluate the clinical efficacy and safety of pm-Pac in combination with gemcitabine as first-line treatment for patients with mPC. The study was performed at the First Affiliated Hospital with Nanjing Medical University, Nanjing, China (the principal clinical research site). The study comprised four phases: screening, treatment, end-of-treatment visit, and follow-up. This study adhered to the principles of the Declaration of Helsinki and was approved by the Ethics Committee of the First Affiliated Hospital with Nanjing Medical University (approval number: 2022-SR-437). All subjects provided written informed consent prior to enrolment. This study was registered at www.chictr.org.cn (identifier: ChiCTR2300078861).

### Patient selection

Eligible patients met the following key inclusion criteria: histologically or cytologically confirmed mPC, no previous systemic treatment of mPC, good performance status with an Eastern Cooperative Oncology Group (ECOG) score of ≤2, measurable disease, normal major organ function, and expected survival of ≥3 months. The key exclusion criteria were as follows: allergy to the study drugs or their components, severe uncontrolled medical conditions, active infections, and previous treatment with paclitaxel or other taxane-based therapies resulting in severe adverse reactions.

### Study procedures

**Figure 1:**
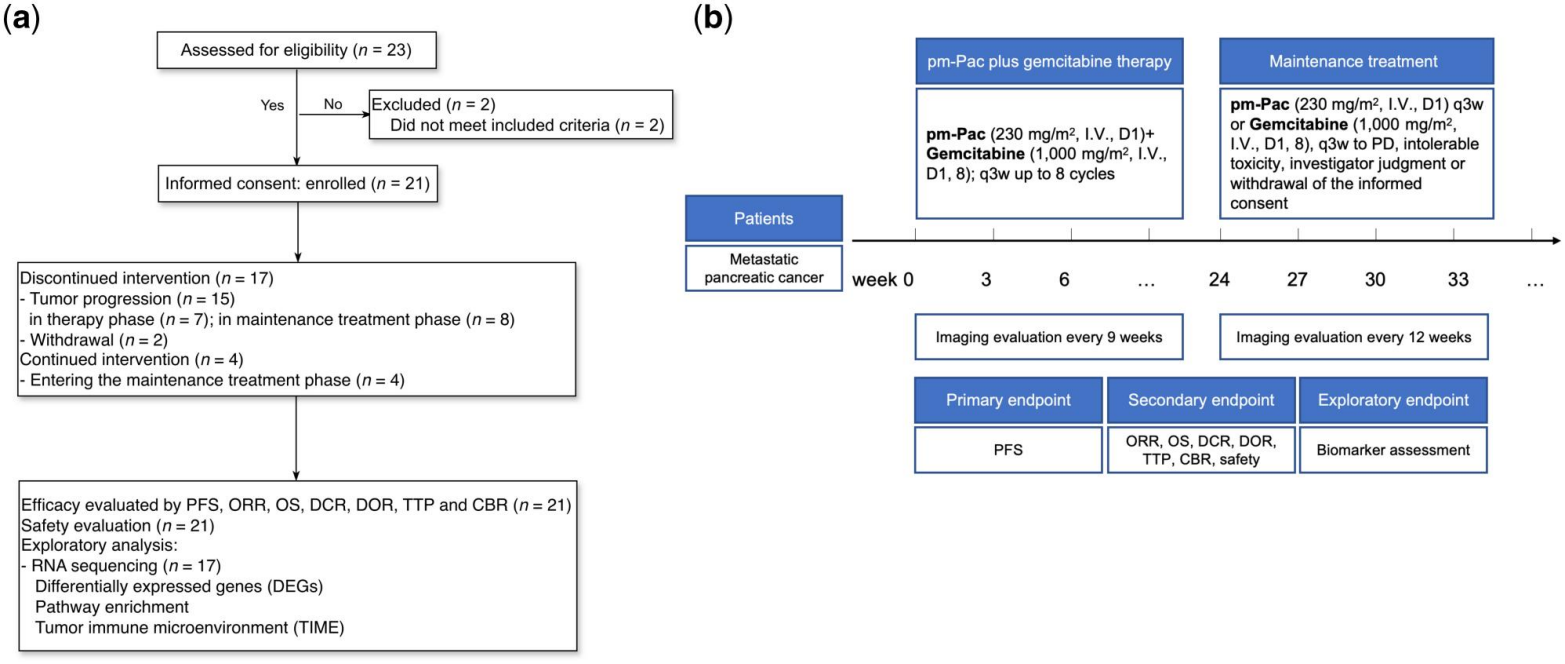
Study flow diagram. (a) Patient dispositions. Twenty-three participants were screened for eligibility and 21 were enrolled. The treatment efficacy was evaluated by using the mPFS, ORR, mOS, disease control rate (DCR), duration of response (DOR), time to progression (TTP), and clinical benefit rate (CBR) (full analysis set, *n *= 21). Safety evaluation was performed (*n *= 21). (b) Timeline diagram showing the treatment regimen tested with eight cycles of pm-Pac plus gemcitabine until progression or intolerable toxicity.

Study drugs and administration: pm-Pac (Shanghai Yizhong Pharmaceutical Co., Ltd) was intravenously administered over 3 h at a dose of 230 mg/m^2^ every 3 weeks. Combination drug: gemcitabine hydrochloride for injection (Eli Lilly) was administered at a dose of 1,000 mg/m^2^ on Days 1 and 8 of each 3-week cycle. The eligible subjects received eight cycles of pm-Pac plus gemcitabine therapy. After eight cycles, patients with complete response (CR), partial response (PR), or stable disease (SD) continued to receive pm-Pac or gemcitabine maintenance treatment at the investigators’ discretion. However, patients with progressive disease (PD) were withdrawn from the study. The reasons for treatment termination included intolerable toxicity, PD, investigators’ judgment, pregnancy, and withdrawal of informed consent. For subjects who discontinued treatment because of non-disease progression/death, follow-up for tumour progression and safety assessments were continued post-treatment (Fig. 1).

### Assessments

Tumour responses were assessed by using computed tomography with contrast every 9 weeks (range, 8–10 weeks) during pm-Pac plus gemcitabine therapy. The maintenance treatment was evaluated every 12 weeks (range, 11–13 weeks). Clinical responses were assessed according to Response Evaluation Criteria in Solid Tumours v1.1 [17]. Safety assessments included physical examinations and laboratory evaluations before each treatment cycle. Adverse events (AEs) were coded by using the Common Terminology Criteria for Adverse Events v5.0. pm-Pac and gemcitabine dose modifications were permitted to manage treatment-related AEs (TRAEs).

### Outcomes

The primary endpoint was PFS, defined as the time from enrolment to PD or death, whichever occurred first. The secondary endpoints included ORR, defined as the proportion of patients achieving a confirmed CR or PR; OS, defined as the time from enrolment to death from any cause; disease control rate (DCR), defined as the proportion of patients with a best overall response of CR, PR, or SD; duration of response (DOR), defined as the time from first documented response to disease progression or death; time to progression (TTP), defined as the time from enrolment to disease progression; clinical benefit rate (CBR), defined as the proportion of patients with CR, PR, or SD lasting for ≥6 months; and safety.

### Biomolecular exploration

Baseline tumour-tissue specimens were collected for biomolecular analysis as required. Formalin-fixed, paraffin-embedded tumour samples were obtained from 17 patients whose tissues were available before treatment. The RNA sequencing details are available in the Supplementary Materials and Methods.

### Statistical analysis

Statistical analyses included all patients who received at least one dose of the study treatment. Continuous variables are presented as means and standard deviations if normally distributed or as medians otherwise. Meanwhile, categorical variables are presented as frequencies. The ORR, CBR, and DCR were estimated by using 95% confidence intervals (CIs) [18]. The Kaplan–Meier method was used to calculate the median times and 95% CIs for time-related events (PFS, DOR, and TTP). All statistical analyses were performed by using SPSS version 26.0 (LEAD Technologies, Inc., Chicago, IL, USA). Statistical analysis of RNA sequencing data is provided in the Supplementary Materials and Methods.

### Data availability

The data generated in this study are available upon request from the corresponding author.

## Results

### Baseline characteristics of patients

**Figure 2:**
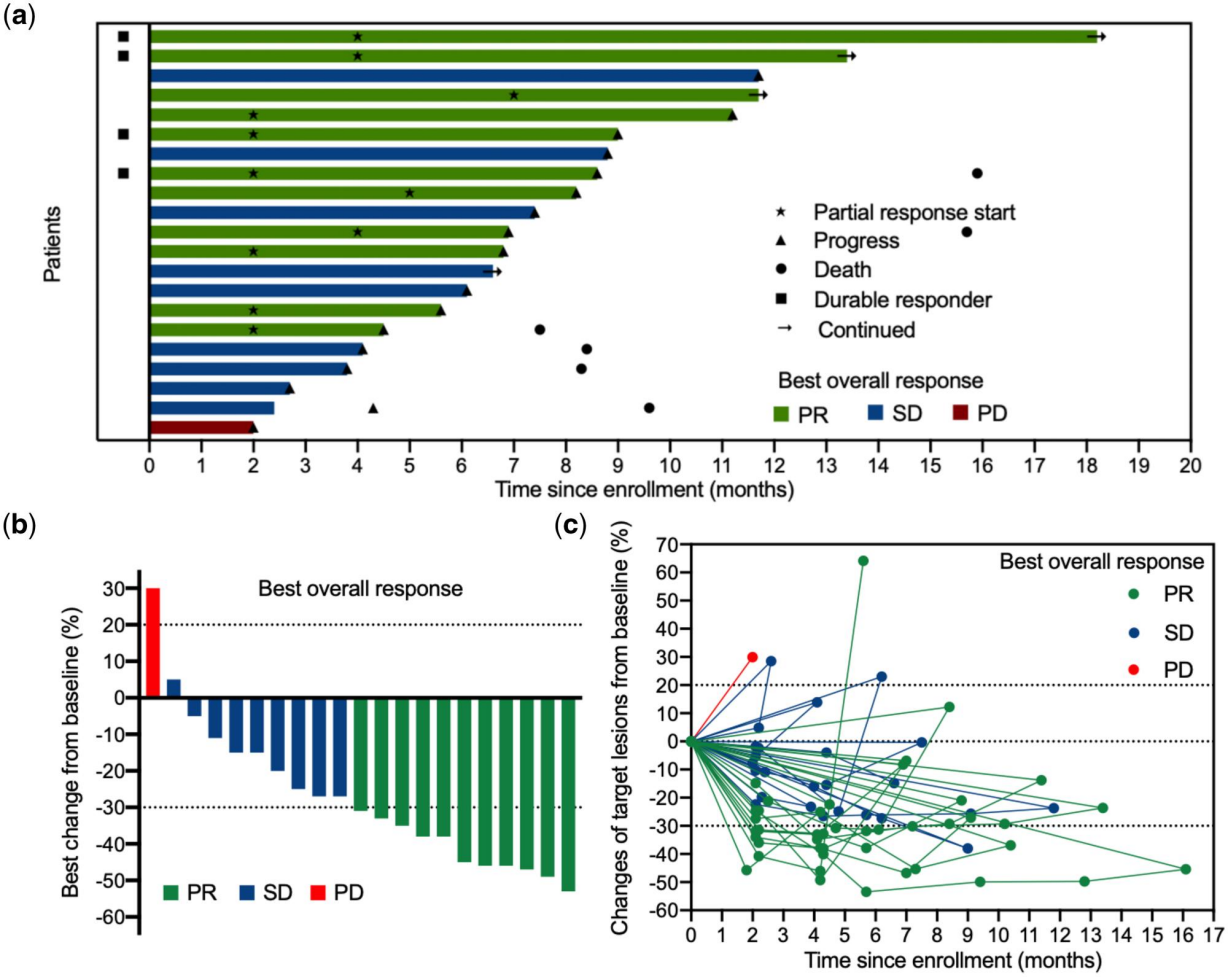
Efficacy of pm-Pac plus gemcitabine. (a) Swimmer plot illustrating PFS for the entire cohort (*n *= 21), with colours indicating the best overall response (BOR). The length of the bars represents the duration from enrolment to PD or withdrawal of informed consent. Death and progression events are represented by circles and triangles, respectively. PR starting time, durable responders (≥6 months), and continued interventions are represented by pentagram, squares, and arrows, respectively. (b) Waterfall plot of best response in target lesion diameter from baseline, with colours indicating BOR (*n *= 21). (c) Spider plot of the change in the target lesion diameter from baseline (*n *= 21).

Twenty-one patients with histologically or cytologically confirmed mPC between April 2023 and May 2024 were enrolled in this study (Fig. 1). Table 1 presents the demographic and clinical characteristics of the patients. The median age of the enrolled patients was 55 years (range, 37–67 years). Amongst them, 14 patients (66.7%) were male. According to the ECOG performance status score, most patients had good performance status, with seven patients (33.3%) having a score of 0, 12 (57.1%) having a score of 1, and two (9.5%) having a score of 2. In terms of body mass index, the majority of patients (85.7%) had a body mass index of <24. Twelve patients (57.1%) had tumours in the head, neck, and uncinate process, and eight patients (38.1%) had tumours in the body/tail. Eleven patients (52.4%) had liver metastasis only, four patients (19.0%) had extrahepatic metastasis only, and six patients (28.6%) had both liver and extrahepatic metastases. The median follow-up period at the data cut-off date (17 December 2024) was 11.8 months. At the time of data cut-off, 21 patients were monitored, with 4 patients (19.0%) undergoing ongoing treatment. Amongst the patients who discontinued treatment (*n *= 17), the reasons for discontinuation included PD (*n *= 15; 88.2%) and withdrawal of informed consent (*n *= 2; 11.8%). Twelve patients entered the maintenance phase and received either pm-Pac or gemcitabine (Fig. 2a). Seven patients (58.3%) were on pm-Pac, whereas five patients (41.7%) were on gemcitabine. Amongst the five patients on gemcitabine, two patients switched to this therapy because of worsened neurotoxicity with prolonged pm-Pac use. These two patients had been on pm-Pac for 7.4 and 9.7 months before transitioning.

**Table 1:** Baseline demographics and clinical characteristics of 21 patients with mPC.

Characteristic	Value
Median age, years (range)	55 (37–67)
≥60	7 (33.3)
Sex, *n* (%)	
Male	14 (66.7)
Female	7 (33.3)
ECOG PS score, *n* (%)	
0	7 (33.3)
1	12 (57.1)
2	2 (9.5)
BMI, *n* (%)	
<24	18 (85.7)
≥24	3 (14.3)
Tumour location, *n* (%)	
Head/neck/uncinate process	12 (57.1)
Body/tail	8 (38.1)
Other	1 (4.8)
Select sites of metastatic disease, *n* (%)	
Liver only	11 (52.4)
Extrahepatic only	4 (19.0)
Liver and extrahepatic	6 (28.6)
Baseline CA19-9, U/mL, *n* (%)	
>30	16 (76.2)
≤30	5 (23.8)
Baseline CA125, U/mL, *n* (%)	
>24	13 (61.9)
≤24	8 (38.1)
Baseline CEA, ng/mL, *n* (%)	
>5	6 (28.6)
≤5	15 (57.1)
Baseline AFP, ng/mL, *n* (%)	
>7	0 (0)
≤7	21 (100)
Baseline CA72-4, U/mL, *n* (%)	
≥6.9	1 (4.8)
<6.9	20 (95.2)

PS = performance status; BMI = body mass index; CA19–9 = carbohydrate antigen 19-9; CA125 = carbohydrate antigen 125; CEA = carcinoembryonic antigen; AFP = alpha-fetoprotein.

### Efficacy assessment

**Figure 3:**
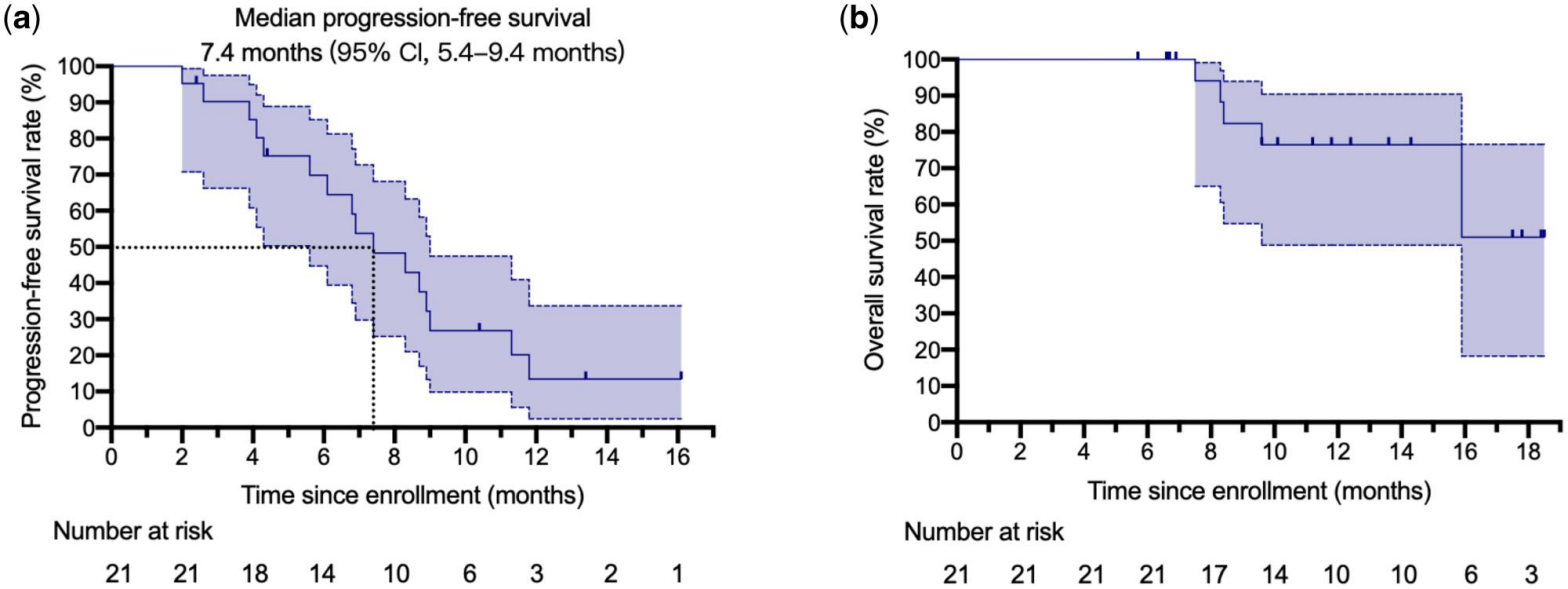
Kaplan–Meier estimation of (a) PFS and (b) overall survival for participants who enrolled in the pm-Pac plus gemcitabine study (full analysis set, *n *= 21). CI, confidence interval.

The efficacy assessment results are presented in Table 2. All 21 patients were eligible for response evaluation and the longest treatment period was 18.2 months. Amongst all patients, 11 patients (52.4%) had PR, 9 patients (42.9%) had SD, and only one patient (4.8%) had PD. The ORR and DCR were 52.4% (95% CI: 29.1%–75.7%) and 95.2% (95% CI: 85.3%–100%), respectively (Fig. 2a–c and Table 2). For patients who achieved PR, the median DOR was 4.8 months (95% CI: 1.5–8.1 months), indicating that these patients had benefited from prolonged disease control. The mPFS was 7.4 months (95% CI: 5.4–9.4 months) (Fig. 3a). Moreover, the 6- and 9-month PFS rates were 66.7% (95% CI: 44.7%–88.7%) and 28.6% (95% CI: 7.5%–49.6%), respectively, providing additional information on long-term survival outcomes. At the cut-off time, 6 out of the 21 patients (28.6%) had died and the mOS had not been reached (Fig. 3b).

**Table 2:** Tumour response and survival outcomes of 21 patients with metastatic pancreatic cancer.

Parameter	Value
Best overall response, *n* (%)	
CR	0 (0)
Partial response	11 (52.4)
Stable disease	9 (42.9)
Progressive disease	1 (4.8)
Not evaluated	0 (0)
ORR, *n* (%) [95% CI]	11 (52.4) [29.1–75.7]
DCR, *n* (%) [95% CI]	20 (95.2) [85.3–100]
CBR, *n* (%) [95% CI]	16 (76.2) [56.3–96.1]
DOR	
Ongoing response, *n*/*N* (%)	11/21 (52.4)
Median (95% CI), months	4.8 (1.5–8.1)
TTP	
Median (95% CI), months	7.4 (5.4–9.4)
PFS	
Events, *n*/*N* (%)	17/21 (81.0)
Median (95% CI), months	7.4 (5.4–9.4)
6-month survival rate, % (95% CI)	66.7 (44.7–88.7)
9-month survival rate, % (95% CI)	28.6 (7.5–49.6)

### Safety assessment

All 21 patients who were enrolled in this study were included in the intention-to-treat population. As shown in Table 3, all patients had TRAEs during therapy and 17 patients (81.0%) had TRAEs of grade 3 or higher. The most common TRAEs included anaemia (*n *= 21, 100%), alopecia (*n *= 21, 100%), total protein abnormality (*n *= 21, 100%), leukopenia (*n *= 19, 90.5%), hypoalbuminemia (*n *= 19, 90.5%), neutropenia (*n *= 18, 85.7%), neurotoxicity (*n *= 17, 81%), and anorexia (*n *= 17, 81%) (Table 3). The most frequent grade 3 or 4 TRAEs included increased γ-glutamyltransferase (GGT) levels (*n *= 8, 38.1%), neutropenia (*n *= 7, 33.3%), and leukocytopenia (*n *= 6, 28.6%). Grade 5 TRAEs were not observed. Treatment-related serious adverse events (SAEs) were observed in three patients, including two patients with febrile neutropenia and one patient with hypokalaemia attributed to the chemotherapy.

**Table 3:** Summary of AEs attributed to pm-Pac plus gemcitabine therapy.

Adverse event	Total	Grade 1	Grade 2	Grade 3	Grade 4	Grade 5
Any treatment-related adverse event	21 (100)	21 (100)	21 (100)	15 (71.4)	5 (23.8)	0 (0)
Grades 3–5 treatment-related AE	17 (81.0)					
Serious adverse event	7 (33.3)					
Treatment-related serious adverse event	3 (14.3)					
Anaemia	21 (100)	21 (100)	15 (71.4)	2 (9.5)	0 (0)	0 (0)
Leukopenia	19 (90.5)	16 (76.2)	14 (66.7)	4 (19)	2 (9.5)	0 (0)
Neutropenia	18 (85.7)	13 (61.9)	15 (71.4)	6 (28.6)	2 (9.5)	0 (0)
Lymphopenia	16 (76.2)	16 (76.2)	7 (33.3)	0 (0)	0 (0)	0 (0)
Thrombocytopenia	12 (57.1)	11 (52.4)	4 (19)	1 (4.8)	0 (0)	0 (0)
Hypoalbuminemia	19 (90.5)	19 (90.5)	1 (4.8)	0 (0)	0 (0)	0 (0)
Total protein abnormality	21 (100)	21 (100)	1 (4.8)	0 (0)	0 (0)	0 (0)
Alanine aminotransferase increased	13 (61.9)	13 (61.9)	2 (9.5)	1 (4.8)	0 (0)	0 (0)
Alkaline phosphatase increased	13 (61.9)	13 (61.9)	4 (19)	0 (0)	0 (0)	0 (0)
Aspartate aminotransferase increased	10 (47.6)	10 (47.6)	1 (4.8)	0 (0)	0 (0)	0 (0)
γ-Glutamyltransferase increased	12 (57.1)	10 (47.6)	9 (42.9)	8 (38.1)	1 (4.8)	0 (0)
Hyperlipidaemia	8 (38.1)	8 (38.1)	0 (0)	0 (0)	0 (0)	0 (0)
Hyponatraemia	8 (38.1)	8 (38.1)	0 (0)	0 (0)	0 (0)	0 (0)
Hyperkalaemia	1 (4.8)	1 (4.8)	0 (0)	0 (0)	0 (0)	0 (0)
Hypokalaemia	6 (28.6)	5 (23.8)	1 (4.8)	0 (0)	1 (4.8)	0 (0)
Anorexia	17 (81)	8 (38.1)	11 (52.4)	1 (4.8)	0 (0)	0 (0)
Constipation	16 (76.2)	15 (71.4)	1 (4.8)	0 (0)	0 (0)	0 (0)
Diarrhoea	7 (33.3)	5 (23.8)	2 (9.5)	0 (0)	0 (0)	0 (0)
Nausea	10 (47.6)	5 (23.8)	6 (28.6)	1 (4.8)	0 (0)	0 (0)
Vomiting	4 (19)	2 (9.5)	1 (4.8)	1 (4.8)	0 (0)	0 (0)
Neurotoxicity	17 (81)	9 (42.9)	14 (66.7)	3 (14.3)	0 (0)	0 (0)
Alopecia	21 (100)	0 (0)	21 (100)	0 (0)	0 (0)	0 (0)
Rash	2 (9.5)	0 (0)	2 (9.5)	0 (0)	0 (0)	0 (0)
Proteinuria	17 (81)	17 (81)	0 (0)	0 (0)	0 (0)	0 (0)
Haematuria	10 (47.6)	10 (47.6)	0 (0)	0 (0)	0 (0)	0 (0)
Pyuria	8 (38.1)	8 (38.1)	0 (0)	0 (0)	0 (0)	0 (0)
Febrile neutropenia	2 (9.5)	0 (0)	0 (0)	0 (0)	2 (9.5)	0 (0)

All data are presented as number of patients followed by percentage in parentheses.

### Exploratory analysis

Transcriptional profiling was performed on baseline tumour-tissue specimens from 17 patients to investigate the potential mechanisms and biomarkers associated with treatment efficacy. The patients were categorized into the long-PFS and short-PFS groups based on an mPFS of 7.4 months. One patient who had not experienced disease progression and had been followed up for <7.4 months was excluded because of insufficient follow-up duration to determine the PFS status. Consequently, 16 patients (9 in the long-PFS group and 7 in the short-PFS group) were included in the comparative analysis. Differentially expressed genes (DEGs), pathway enrichment, and the tumour immune microenvironment (TIME) were assessed between the two groups.

**Figure 4:**
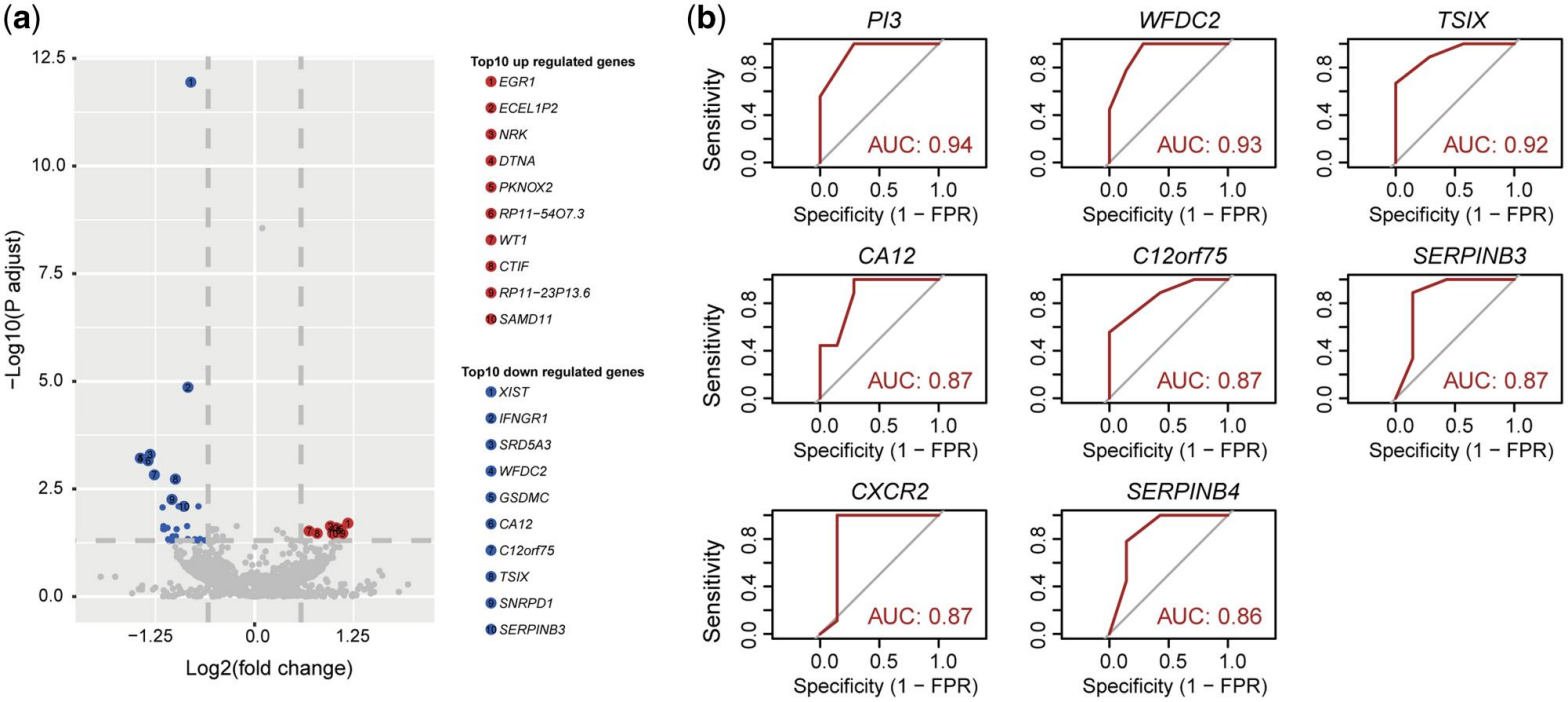
Differential gene-expression analysis between patients with long and short PFS. (a) Volcano plot of differential gene expression between long-PFS (≥7.4 months) and short-PFS (<7.4 months) groups. Vertical and horizontal dashed lines indicate ±log_2_ (fold change) and –log_10_ (false discovery rate) thresholds, respectively. Top up- and downregulated genes are labelled. (b) Receiver-operating characteristic curves for eight differentially expressed candidate genes, with corresponding area-under-the-curve values demonstrating strong discrimination between PFS groups.

Differential gene-expression analysis identified 49 DEGs between patients with long and short PFS, including 15 upregulated genes in the long-PFS group and 34 upregulated genes in the short-PFS group (Fig. 4a and Supplementary Table S1). Amongst them, the expression of eight genes (e.g. WFDC2, PI3, C12orf75, TSIX, CA12, SERPINB4, SERPINB3, and CXCR2) showed statistically significant differences between the two groups, as determined by using the Wilcoxon rank-sum test (false discovery rate <0.1, Supplementary Table S1). To further assess the predictive value of the identified DEGs, receiver-operating characteristic curve analysis was performed. Eight DEGs exhibited strong discriminative performance, each with area-under-the-curve values of >0.85 (Fig. 4b).

**Figure 5:**
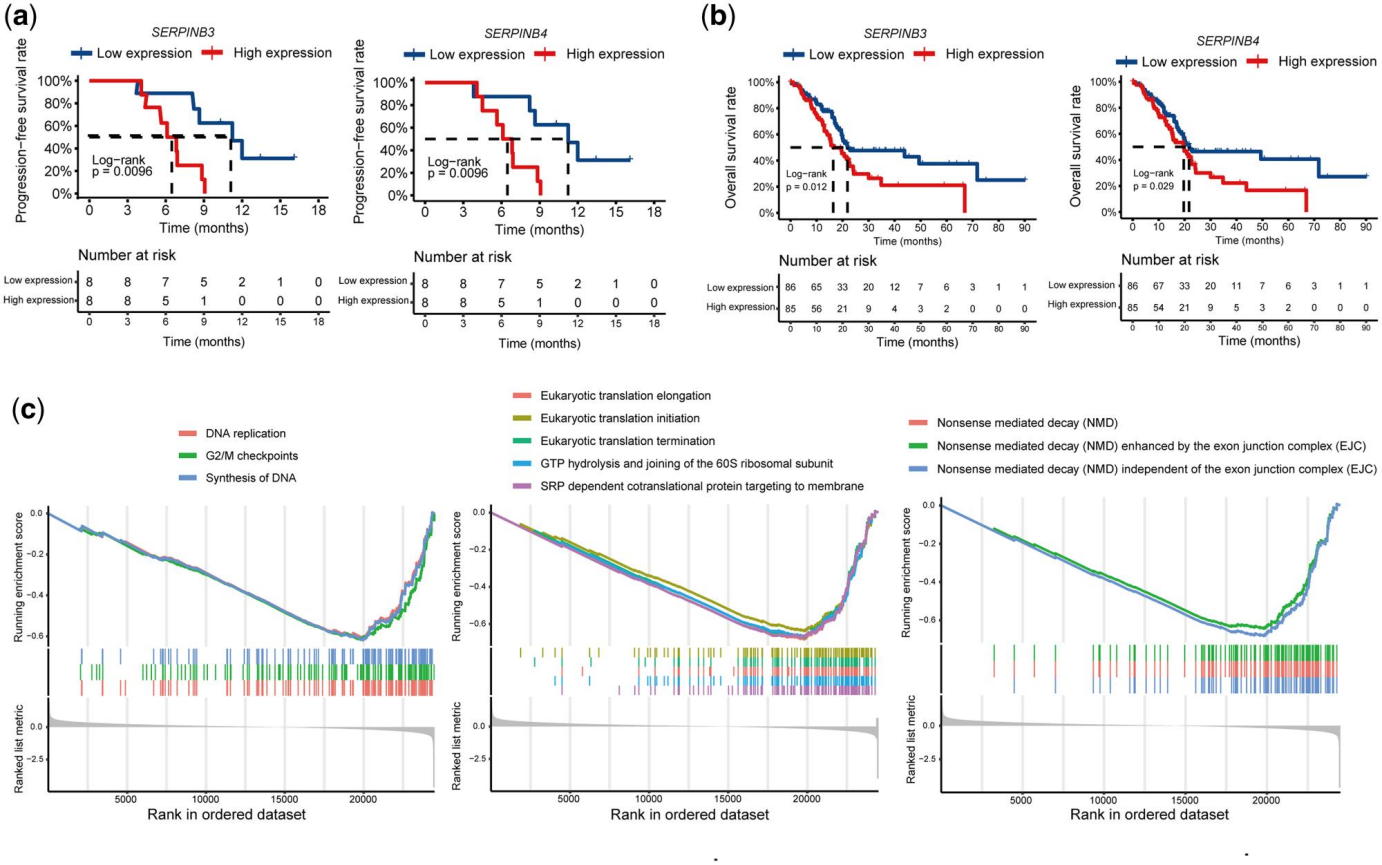
Integrated transcriptomic analysis associated with PFS. (a) Kaplan–Meier curves for PFS stratified by high versus low expression of two representative DEGs. The log-rank *P* values are indicated. (b) Kaplan–Meier curves for overall survival similarly stratified by the same two genes. (c) Representative pre-ranked gene set enrichment analysis (GSEA) plots for Reactome pathways significantly enriched in the long-PFS group, including DNA replication, nonsense-mediated decay, and protein translation.

By incorporating patient survival data to assess the prognostic impact of these DEGs, we found that patients with low WFDC2, CXCR2, SERPINB3, and SERPINB4 expression had significantly longer PFS than those with high expression (Fig. 5a and Supplementary Fig. S1). To validate these observations in an independent cohort, we analysed the TCGA-PAAD dataset (*n *= 171). Consistently, low SERPINB3 and SERPINB4 expression was associated with markedly improved OS relative to high expression (Fig. 5b). This suggests that decreased SERPINB3 and SERPINB4 levels may predict a more favourable prognosis and enhance patient survival.

We performed gene set enrichment analysis (GSEA) to further explore the biological processes underlying the treatment response. Compared with those in the short-PFS group, tumours in the long-PFS group demonstrated significant downregulation of the gene sets involved in DNA replication, nonsense-mediated mRNA decay (NMD), and protein translation (Fig. 5c). This coordinated suppression suggests that inhibiting these fundamental processes may sensitize tumour cells to chemoradiotherapy and contribute to improved therapeutic outcomes.

**Figure 6:**
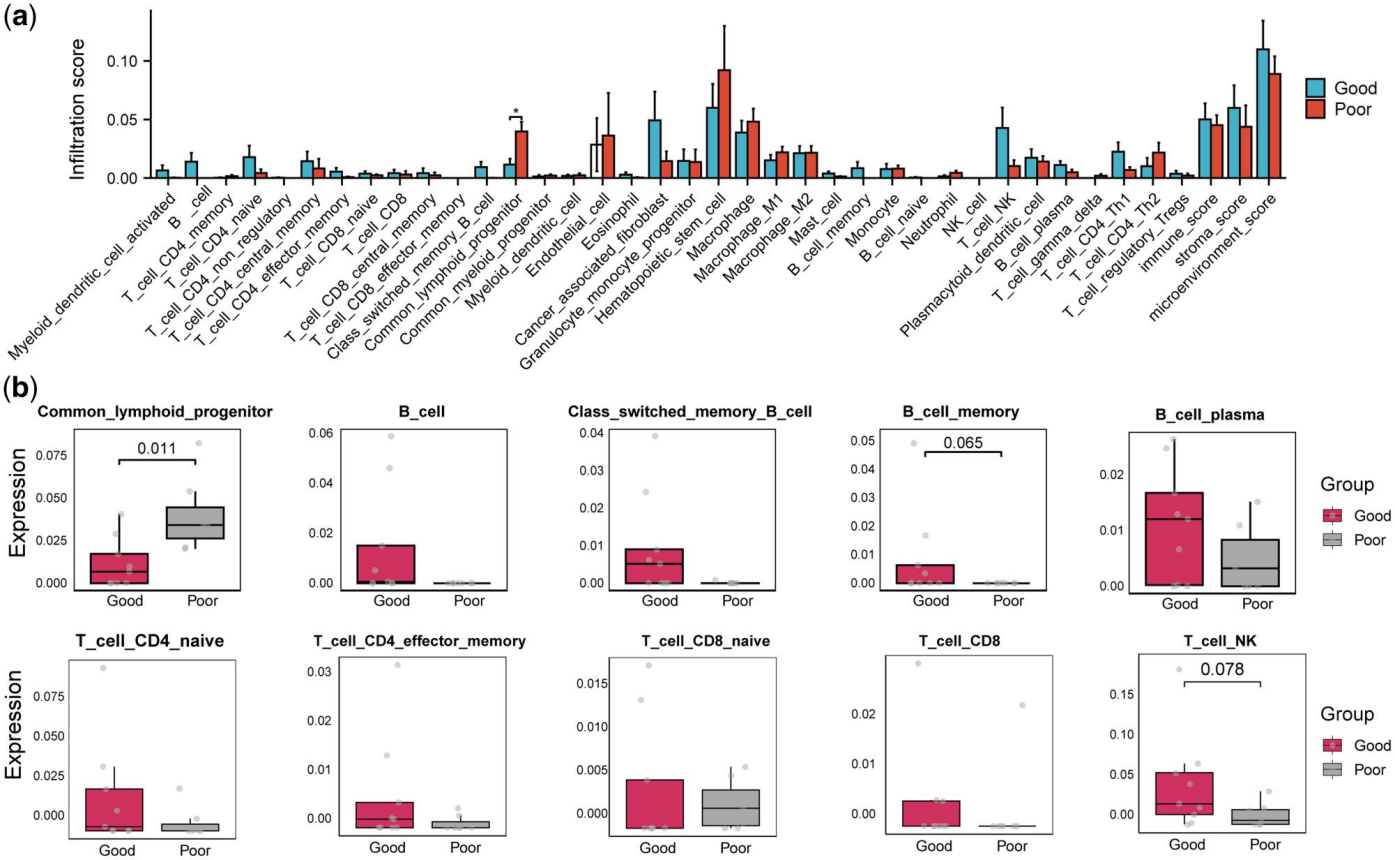
Tumour immune microenvironment analysis associated with PFS. (a) xCell-derived relative proportions of immune cell types across PFS groups. The bar height represents the relative proportions for each cell type in long-PFS (good) versus short-PFS (poor) tumours. (b) Box plots of selected lymphoid subpopulations, including common lymphoid progenitors (CLPs), B-cell subsets, CD4^+^ memory T cells, and natural killer (NK) cells, showing differences in enrichment between the PFS groups.

Finally, we applied xCell deconvolution to characterize the TIME. Compared with the long-PFS group, the short-PFS group showed significantly higher enrichment of common lymphoid progenitors (*P *< 0.05) and pronounced reductions in B- and T-cell populations, including naive and memory subsets (Fig. 6). The combination of elevated progenitor levels and depleted mature lymphocytes suggests a block in lymphoid differentiation, which may weaken adaptive immune surveillance and cytotoxic function, foster an immunosuppressive microenvironment, and limit the therapeutic efficacy.

## Discussion

The high mortality rate and limited therapeutic options for pancreatic cancer highlight the need to explore novel treatment strategies. As an exploratory trial, this study demonstrated that the combination therapy of pm-Pac and gemcitabine, administered as first-line treatment for mPC, exhibited favourable tolerability and promising clinical efficacy. In this study, patients treated with the pm-Pac and gemcitabine combination regimen achieved an mPFS of 7.4 months. The ORR, DCR, and median DOR were 52.4%, 95.2%, and 4.8 months, respectively. Although direct comparisons with established regimens, including AG (mPFS, 5.5–5.6 months), FOLFIRINOX (mPFS, 6.4–7.4 months), and NALIRIFOX (mPFS, 6.4–7.4 months), are limited by the small sample size, single-arm design, and non-randomized approach, these preliminary findings may serve as a hypothesis-generating foundation for subsequent large-scale randomized–controlled trials [10–12]. Moreover, the pm-Pac and gemcitabine combination regimen offers an alternative option, particularly for patients who cannot tolerate more intensive regimens because of poor performance status or comorbidities.

With regard to safety outcomes, although 81.0% of the patients experienced grade ≥3 AEs, the overall profile of these AEs was generally consistent with that reported in two previous phase III trials of AG combination therapy [10, 12]. The most common grade ≥3 AEs in our study were neutropenia (33.3%), leukopenia (28.6%), and GGT elevation (38.1%). The incidence of SAEs was 33.3%, with treatment-related SAEs occurring in 14.3% of patients. No treatment-related deaths were reported. In the NAPOLI-3 trial, SAEs, treatment-related SAEs, and treatment-related deaths were reported in 52%, 19%, and 2% of patients, respectively. Meanwhile, the MPACT trial reported treatment-related deaths in 4% of patients. Specifically, the incidence of grade ≥3 anaemia (9.5%) and thrombocytopenia (4.8%) was lower than that reported in the AG groups of the NAPOLI-3 trial (17% for anaemia) and MPACT trial (13% for anaemia, 13% for thrombocytopenia). Conversely, we observed a higher rate of GGT elevation (38.1% vs 6% in NAPOLI-3) and febrile neutropenia (9.5% vs 3% in MPACT). The incidence of neurotoxicity (14.3%) fell between the rates reported in MPACT (17%) and NAPOLI-3 (6%). These findings suggest that, although the pm-Pac formulation maintains a comparable overall toxicity profile to AG regimens, it demonstrates a distinct pattern characterized by an increased signal of hepatotoxicity. The numerically lower rates of SAEs, treatment-related SAEs, and fatal AEs, along with a more favourable haematologic toxicity profile (particularly for anaemia and thrombocytopenia) may indicate a potentially improved safety profile in terms of severe complications. However, the elevated hepatotoxicity warrants careful monitoring in clinical practice.

Furthermore, the treatment schedule of pm-Pac (administered every 3 weeks [on Day 1]) in combination with gemcitabine (on Days 1 and 8 of a 21-day cycle) was selected based on pharmacological rationale and alignment with clinical guidelines and real-world practice. The 3-week dosing interval is supported by the established pharmacokinetic profile of pm-Pac and has been validated in previous clinical trials, including a phase I study in advanced solid tumours that demonstrated good tolerability up to 390 mg/m^2^ and a phase III trial in NSCLC in which pm-Pac showed improved efficacy and safety compared with solvent-based paclitaxel [15, 16]. Furthermore, this regimen aligns with the modified gemcitabine-based schedules recommended by the Chinese Society of Clinical Oncology Pancreatic Cancer Guidelines (2024 edition), which recommend administration on Days 1 and 8 of a 21-day cycle [19]. Compared with the standard AG regimen (Days 1, 8, and 15 every 28 days), the current schedule reduces hospital visits from three to two per cycle, thereby enhancing patient convenience and potentially improving treatment adherence and quality of life. The efficacy, safety, and patient-centred design of the pm-Pac and gemcitabine combination regimen support its role as a valuable first-line option for mPC.

In our exploratory analysis, eight genes could robustly discriminate between patients with long and short PFS. In particular, low SERPINB3 and SERPINB4 expression was associated with prolonged PFS. SERPINB3 (also known as SCCA1) and SERPINB4 (SCCA2) are intracellular serine protease inhibitors that block cathepsin L, K, and S, thereby preventing apoptosis in carcinomas, including pancreatic cancer. Elevated expression of these inhibitors has been shown to promote extracellular matrix remodelling and activate pro-survival signalling pathways that enable tumour cells to withstand cytotoxic stress [20–22]. SERPINB3, which is highly expressed in the basal-like/squamous subtype of pancreatic cancer, is associated with poor survival outcomes. It activates the oncogene MYC by inhibiting calpain—a cysteine protease involved in the MYC degradation pathway—thus driving cancer-associated metabolic reprogramming [23]. Additionally, SERPINB3 can induce epithelial–mesenchymal transition-like phenotypes, promoting migration, therapeutic resistance, and proliferation [21]. SERPINB4, which shares high homology with SERPINB3, acts as a critical downstream regulator of the Ras/MAPK pathway in RAS-driven tumour models, enhancing pro-inflammatory cytokine production and facilitating pancreatic tumour progression [22]. Our finding that reduced SERPINB3 and SERPINB4 expression is correlated with better outcomes suggests that inhibiting their activity, either directly or by targeting upstream regulators, could sensitize tumours to DNA-damaging treatments.

Second, GSEA revealed that long-PFS tumours exhibited significant downregulation of gene sets involved in DNA replication (e.g. S-phase synthesis and G_2_/M checkpoint control), NMD, and protein translation. DNA replication drives cell proliferation. Therefore, suppressing DNA replication results in a lower rate of tumour-cell division and fewer opportunities for replication-induced DNA damage. NMD is an mRNA quality-control pathway that relies on the exon junction complex to recognize and degrade transcripts carrying premature stop codons, thereby preventing truncated protein synthesis. As reported in UPF1-mutant pancreatic tumours, NMD attenuation may increase the burden of aberrant peptides and boost tumour immunogenicity [24, 25]. The reduced expression of protein translation machinery genes points to a diminished capacity for protein synthesis, further constraining tumour growth. Together, the coordinated suppression of these fundamental processes may sensitize tumour cells to chemoradiotherapy by limiting their replicative potential and promoting the apoptotic clearance of damaged cells.

Third, TIME analysis revealed that patients with short PFS harbour increased levels of common lymphoid progenitors along with sharply decreased mature B- and T-lymphocyte populations, which points to a differentiation blockade that undermines adaptive immunity. By contrast, the maintenance of memory T cell and natural killer (NK) cell populations in the long-PFS group suggests that the preservation of innate and adaptive effector compartments is critical for tumour control [26]. These insights highlight the potential of combining cytotoxic chemotherapy with interventions that restore lymphoid differentiation or enhance effector functions, including cytokine therapies or checkpoint blockade, to reshape the tumour microenvironment and improve patient outcomes.

Collectively, the findings from our transcriptomic and immunophenotypic exploratory analyses provide novel insights into the molecular and cellular underpinnings of treatment-response variability. However, it is important to acknowledge the exploratory nature of these results. Future studies with larger, independent patient cohorts are needed to validate the prognostic value of the identified gene signatures and associated biological pathways, and to confirm the immunologic correlations of the prolonged PFS described herein.

This study has several limitations that should be considered when interpreting the results. First, the single-arm design and limited sample size constrain the robustness and generalizability of the conclusions. Second, the lack of a randomized control group and blinded independent central review for tumour-response assessment means that the efficacy findings should be interpreted with caution because of potential biases. Third, the study population predominantly comprised patients with good performance status, which may have led to selection bias and limit the applicability of the results to a broader, more unselected patient population with mPC. Therefore, although the current findings demonstrate promising efficacy and a manageable safety profile, they need to be validated through larger-scale, randomized–controlled trials with long-term follow-up.

## Conclusions

pm-Pac combined with gemcitabine as first-line therapy for mPC exhibits favourable tolerability and promising clinical efficacy. However, these preliminary findings require further validation in larger randomized–controlled trials. In future studies, we will continue to explore the efficacy and safety of this treatment regimen in a broader patient population and perform comparative analyses with other first-line treatment options to provide better therapeutic choices for patients with mPC.

## Supplementary Material

goag034_Supplementary_Data
