# Supplementary material for: First-line nanoparticle polymeric micellar paclitaxel with gemcitabine in metastatic pancreatic cancer: a single-arm, prospective, and exploratory study
Source: Gastroenterol Rep (Oxf). 2026 May 1;14:goag034. doi: 10.1093/gastro/goag034 (PMC13132657; doi:10.1093/gastro/goag034)
Supplement: goag034_Supplementary_Data [file goag034_supplementary_data.zip › 2025-470 Supplementary materials and methods.docx]

**1. Supplementary materials and methods**

1.1. RNA sample cohort and collection

Total RNA was successfully extracted and passed quality control in 17 of the 19 patients; two samples failed due to insufficient yield or degradation and were excluded. During grouping, one additional patient whose follow-up duration was shorter than the cohort median PFS of 7.4 months was omitted. The final evaluable cohort comprised 16 patients, of whom nine were assigned to the long-PFS group (≥ 7.4 months) and seven to the short-PFS group (< 7.4 months).

1.2. Differential expression analysis (DEG)

Total RNA was extracted from FFPE tumor and assessed for quality on an Agilent 2100 Bioanalyzer. RNA-seq libraries were prepared using the KAPA Stranded RNA-Seq Kit with RiboErase (KAPA Biosystems), and paired-end sequencing was performed on an Illumina HiSeq4000 platform, generating an average of 60M reads per sample. Raw sequencing data were processed using the FastQC tool for quality control, followed by trimming of adapter sequences using Trimmomatic. The cleaned reads were aligned to the human reference genome (GRCh38) using STAR aligner, and gene expression levels were quantified using featureCounts. Differential gene expression analysis was conducted using the DESeq2 package, and DEGs were defined by an absolute fold change ≥ 1.5 and a Benjamini–Hochberg–adjusted p-value (padj) < 0.05. For complementary nonparametric confirmation, Wilcoxon rank-sum tests on gene expression counts were also carried out, with FDR < 0.10 indicating significance.

1.3. Receiver operating characteristic (ROC) analysis

DEGs produced by DESeq2 were exported into R (v4.1.2) for predictive performance assessment. ROC curves were constructed using the pROC package, with expression thresholds set at the point maximizing Youden’s index to balance sensitivity and specificity. The area under each ROC curve (AUC) was then computed, and genes achieving an AUC > 0.85 were classified as strong discriminators of long- versus short-PFS groups.

1.4. External validation in TCGA cohort

Publicly available RNA-seq and clinical data for pancreatic ductal adenocarcinoma (PAAD) were downloaded from The Cancer Genome Atlas (TCGA) (paad_tcga_pan_can_atlas_2018, *n* = 171). Patients were divided into high- and low-expression groups for SERPINB3 and SERPINB4 based on median gene expression counts. Kaplan-Meier survival curves for PFS and overall survival (OS) were plotted, and differences assessed by the log-rank test.

1.5. Gene set enrichment analysis (GSEA)

Pre-ranked gene set enrichment analysis was carried out using the clusterProfiler package against Reactome pathway definitions obtained from MSigDB. Genes were ordered by the signed–log_10_ (p-value) from the Wilcoxon rank-sum test, and enrichment was assessed over 1,000 phenotype permutations. Only gene sets comprising 10 to 1,000 members were considered. Pathways with a nominal p-value < 0.05 and Benjamini-Hochberg-adjusted p-value < 0.25 were deemed significantly enriched, and the twenty highest-ranking pathways by normalized enrichment score were reported.

1.6. Tumor immune microenvironment analysis

The tumor immune microenvironment was characterized by estimating immune cell infiltration with the xCell algorithm (25) via the TIMER2.0 web portal (http://timer.cistrome.org/). For each tumor specimen, we used xCell to infer the relative abundance of diverse immune cell populations. These inferred cell proportions were then compared between patients with long versus short PFS using the Wilcoxon rank-sum test, and multiple testing was controlled by the false discovery rate (FDR) method.

1.7. Statistical analysis

All statistical analyses were conducted in R (v4.1.2). Continuous variables were compared by Wilcoxon rank-sum test; categorical variables by Fisher’s exact test. Survival analyses employed Kaplan–Meier estimation and log-rank testing. Two-sided *P* < 0.05 was considered statistically significant unless otherwise noted.
