# Supplementary material for: First-line nanoparticle polymeric micellar paclitaxel with gemcitabine in metastatic pancreatic cancer: a single-arm, prospective, and exploratory study
Source: Gastroenterol Rep (Oxf). 2026 May 1;14:goag034. doi: 10.1093/gastro/goag034 (PMC13132657; doi:10.1093/gastro/goag034)
Supplement: goag034_Supplementary_Data [file goag034_supplementary_data.zip › 2025-470 Supplementary Table S1.docx]

Supplementary Table 1: Differential gene expression analysis identified 49 DEGs between patients with long and short PFS

| Gene ID | Gene symbol | Log2 fold change | p value | padj | Wilcox_p | Wilcox_p_adjust |
| --- | --- | --- | --- | --- | --- | --- |
| ENSG00000101443.13 | WFDC2 | -1.44083836 | 0.000000153 | 0.000610386 | 0.002142843 | **0.068124018** |
| ENSG00000235162.4 | C12orf75 | -1.264024004 | 0.000000496 | 0.001485628 | 0.004263431 | **0.068124018** |
| ENSG00000270641.1 | TSIX | -0.998929106 | 0.000000703 | 0.001869541 | 0.005561144 | **0.068124018** |
| ENSG00000124102.4 | PI3 | -1.149427472 | 0.000017 | 0.023101293 | 0.003016848 | **0.068124018** |
| ENSG00000074410.9 | CA12 | -1.342117565 | 0.000000203 | 0.000695882 | 0.008138227 | **0.079754628** |
| ENSG00000057149.10 | SERPINB3 | -0.89106069 | 0.00000405 | 0.008016812 | 0.016343509 | **0.103565377** |
| ENSG00000206073.6 | SERPINB4 | -0.958191061 | 0.00000435 | 0.008016812 | 0.015237759 | **0.103565377** |
| ENSG00000180871.3 | CXCR2 | -1.151405397 | 0.0000258 | 0.026978806 | 0.016908633 | **0.103565377** |
| ENSG00000123572.12 | NRK | 1.030892868 | 0.0000222 | 0.02530392 | 0.019601959 | 0.106721777 |
| ENSG00000128039.6 | SRD5A3 | -1.316172471 | 8.33E-08 | 0.000498458 | 0.026224181 | 0.107082074 |
| ENSG00000167536.9 | DHRS13 | -1.137423381 | 0.0000291 | 0.027216432 | 0.025784472 | 0.107082074 |
| ENSG00000267469.1 | AC005944.2 | -1.033035748 | 0.0000671 | 0.039757237 | 0.026224181 | 0.107082074 |
| ENSG00000101773.12 | RBBP8 | -1.159911188 | 0.00000496 | 0.008478345 | 0.044307158 | 0.127708867 |
| ENSG00000184937.8 | WT1 | 0.687707812 | 0.0000412 | 0.02991987 | 0.041824715 | 0.127708867 |
| ENSG00000244953.1 | RP11-613D13.8 | 1.003047915 | 0.0000794 | 0.042256517 | 0.038720866 | 0.127708867 |
| ENSG00000064655.14 | EYA2 | -1.089634234 | 0.000101228 | 0.046535734 | 0.038720866 | 0.127708867 |
| ENSG00000170006.7 | TMEM154 | -1.024127249 | 0.000110951 | 0.049200718 | 0.044307158 | 0.127708867 |
| ENSG00000147697.4 | GSDMC | -1.437735299 | 0.000000148 | 0.000610386 | 0.049694802 | 0.135280296 |
| ENSG00000167088.6 | SNRPD1 | -1.0420255 | 0.00000231 | 0.005534865 | 0.056738221 | 0.146324887 |
| ENSG00000027697.8 | IFNGR1 | -0.840203983 | 1.72E-09 | 0.0000138 | 0.071944235 | 0.167869883 |
| ENSG00000116711.8 | PLA2G4A | -1.04497116 | 0.000128161 | 0.04950859 | 0.071526063 | 0.167869883 |
| ENSG00000244280.1 | ECEL1P2 | 0.956859865 | 0.0000158 | 0.023101293 | 0.087968664 | 0.17706168 |
| ENSG00000188086.8 | PRSS45 | 1.132265481 | 0.0000552 | 0.033997434 | 0.089863967 | 0.17706168 |
| ENSG00000267454.1 | ZNF582-AS1 | 1.076601704 | 0.0000554 | 0.033997434 | 0.08915337 | 0.17706168 |
| ENSG00000112378.11 | PERP | -0.993045004 | 0.0000681 | 0.039757237 | 0.090337592 | 0.17706168 |
| ENSG00000196611.4 | MMP1 | -0.982963578 | 0.0000304 | 0.027216432 | 0.112074586 | 0.183486586 |
| ENSG00000174171.4 | RP11-23P13.6 | 1.112577423 | 0.0000522 | 0.03389462 | 0.112074586 | 0.183486586 |
| ENSG00000214652.4 | RP11-3N2.13 | 1.104827005 | 0.0000719 | 0.039757237 | 0.112338726 | 0.183486586 |
| ENSG00000160321.10 | ZNF208 | 1.044537289 | 0.0000893 | 0.045964393 | 0.112338726 | 0.183486586 |
| ENSG00000090263.11 | MRPS33 | -0.769043 | 0.000130276 | 0.04950859 | 0.112338726 | 0.183486586 |
| ENSG00000187634.6 | SAMD11 | 0.984079192 | 0.0000523 | 0.03389462 | 0.138362342 | 0.205447114 |
| ENSG00000134049.3 | IER3IP1 | -0.689029672 | 0.000102998 | 0.046535734 | 0.138362342 | 0.205447114 |
| ENSG00000117862.7 | TXNDC12 | -0.630172514 | 0.000114647 | 0.049257766 | 0.138362342 | 0.205447114 |
| ENSG00000229807.5 | XIST | -0.804171108 | 4.71E-17 | 1.13E-12 | 0.152704556 | 0.213786379 |
| ENSG00000085377.9 | PREP | -0.719188939 | 0.000132487 | 0.04950859 | 0.152704556 | 0.213786379 |
| ENSG00000169019.9 | COMMD8 | -0.841658835 | 0.0000941 | 0.045979021 | 0.16848787 | 0.217664737 |
| ENSG00000119314.11 | PTBP3 | -0.751995572 | 0.0000998 | 0.046535734 | 0.168801225 | 0.217664737 |
| ENSG00000132589.11 | FLOT2 | -0.620476979 | 0.000122028 | 0.04950859 | 0.168801225 | 0.217664737 |
| ENSG00000223764.2 | RP11-54O7.3 | 1.05911969 | 0.0000391 | 0.02924605 | 0.185141167 | 0.232613262 |
| ENSG00000165495.11 | PKNOX2 | 1.088164374 | 0.000033 | 0.027216432 | 0.204008164 | 0.243814635 |
| ENSG00000183726.6 | TMEM50A | -0.608954257 | 0.000130632 | 0.04950859 | 0.204008164 | 0.243814635 |
| ENSG00000120738.7 | EGR1 | 1.17803781 | 0.0000124 | 0.01972604 | 0.244276744 | 0.265990232 |
| ENSG00000160131.9 | VMA21 | -0.850856897 | 0.0000181 | 0.023101293 | 0.244276744 | 0.265990232 |
| ENSG00000134030.9 | CTIF | 0.791091218 | 0.0000524 | 0.03389462 | 0.244276744 | 0.265990232 |
| ENSG00000167005.9 | NUDT21 | -0.68673708 | 0.0000902 | 0.045964393 | 0.244276744 | 0.265990232 |
| ENSG00000118849.5 | RARRES1 | -1.098597167 | 0.000022 | 0.02530392 | 0.266028098 | 0.283377756 |
| ENSG00000134769.17 | DTNA | 0.981179568 | 0.0000295 | 0.027216432 | 0.313901757 | 0.327259278 |
| ENSG00000075303.8 | SLC25A40 | -0.708017755 | 0.00000412 | 0.008016812 | 0.340761848 | 0.347861053 |
| ENSG00000187908.11 | DMBT1 | -0.623851953 | 0.00012631 | 0.04950859 | 0.396405748 | 0.396405748 |
