# Supplementary figures and images for: First-line nanoparticle polymeric micellar paclitaxel with gemcitabine in metastatic pancreatic cancer: a single-arm, prospective, and exploratory study
Source: Gastroenterol Rep (Oxf). 2026 May 1;14:goag034. doi: 10.1093/gastro/goag034 (PMC13132657; doi:10.1093/gastro/goag034)

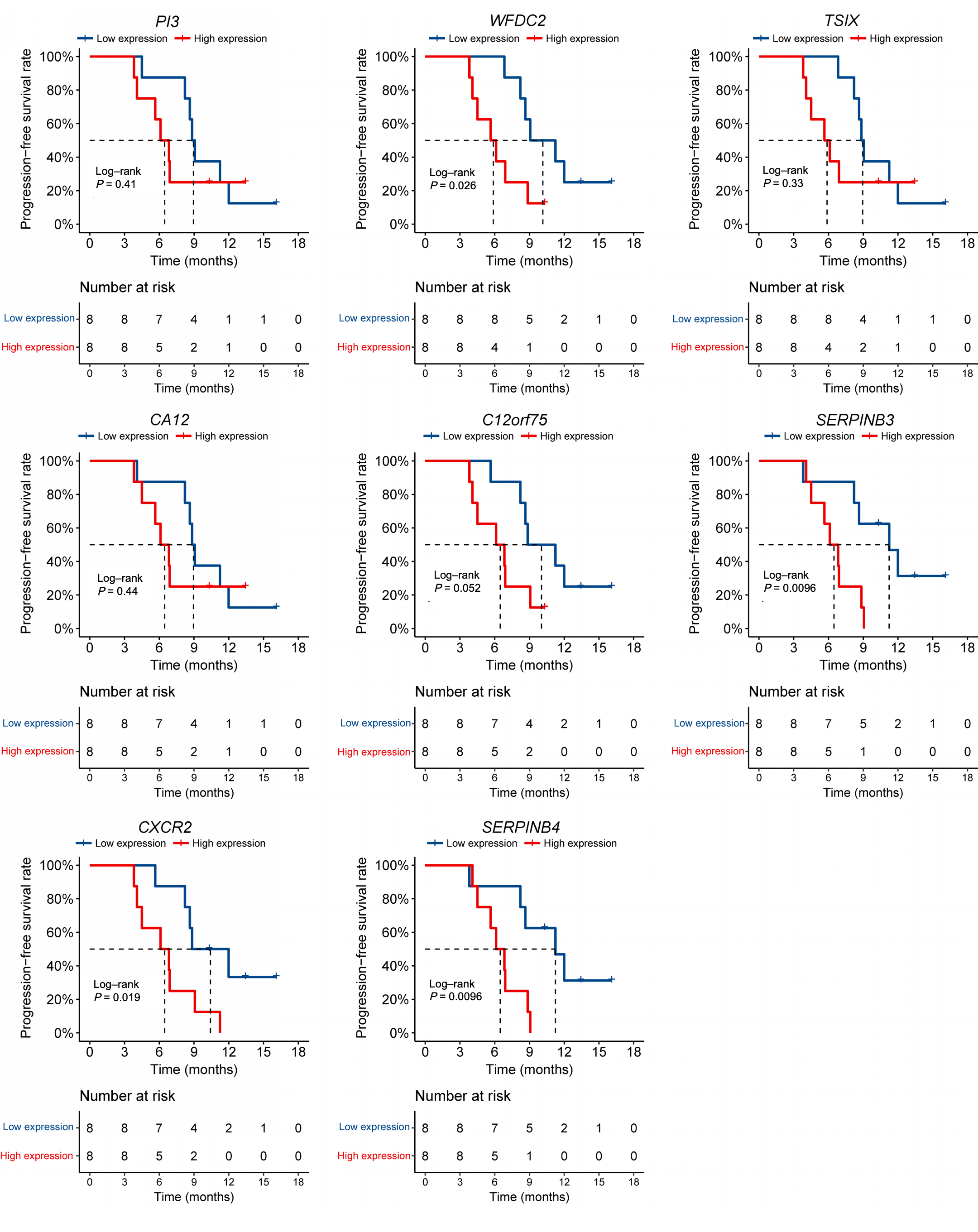

Supplement: goag034_Supplementary_Data [file goag034_supplementary_data.zip › 2025-470 Supplementary Figure S1.tif]
